# Dermoscopy of very small basal cell carcinoma (≤3 mm)^☆^

**DOI:** 10.1016/j.abd.2022.12.004

**Published:** 2023-07-06

**Authors:** Camilo Arias-Rodriguez, Ana Maria Muñoz-Monsalve, Diana Cuesta, Susana Mejia-Mesa, Maria Soledad Aluma-Tenorio

**Affiliations:** a Department of Dermatology, Universidad Pontificia Bolivariana, Medellin, Colombia; b Department of Dermatology, Aurora Center Specialized in Piel Cancer, Medellin, Colombia; c Department of Dermatology, Universidad El Bosque, Bogota, Colombia

**Keywords:** Carcinoma, basal cell, Dermoscopy, Diagnosis, Skin neoplasms

## Abstract

**Background:**

Basal cell carcinoma (BCC) dermoscopy is key to lower the biopsy threshold of suspicious lesions. There is a scarcity of published data on the dermoscopy of very small BCC (≤3 mm) and its differences from larger BCCs.

**Objective:**

To describe and compare dermoscopic features of BCCs measuring ≤3 mm, with those from 3 to 10 mm.

**Methods:**

An analytical cross-sectional study, included biopsy-proven BCCs that had dermoscopic photographic images, between January 2017 and December 2022 in a Skin Cancer Center in Medellín, Colombia. Demographic, clinic-pathological and dermoscopic features were compared between very small BCCs (vsBCCs) and a reference group.

**Results:**

A total of 326 BCCs in 196 patients were included, of whom 60% were male. The most common Fitzpatrick phototype was III. vsBCCs accounted for 25% of the lesions (81/326). Face and neck were the most frequent locations (53%), especially in very small tumors. The nodular type was more common in very small tumors than in larger lesions, the superficial type was less frequent, and aggressive types were equally prevalent in both groups. On dermoscopy, very small tumors were statistically more likely to present pigmented structures than reference lesions, especially blue-gray dots (67% vs. 54%), vessels were less frequent, particularly short-fine telangiectasias (SFT) (52% vs. 66%), as were other structures such as shiny white structures (SWS), ulceration, micro-erosions, and scales.

**Study limitations:**

Latin-American sample, lacks information on dark phototypes

**Conclusions:**

Pigmented structures, especially blue-gray dots, were most common in vsBCCs when compared to larger lesions; SFT, SWS and other findings were less prevalent.

## Introduction

Basal Cell Carcinoma (BCC) is the most common skin cancer worldwide, with an incidence so high, that it is out of global cancer statistics.^1^ Over the last decades, its incidence has risen, and despite its low lethality, it can have an aggressive local behavior and it places a high burden on global health systems.^2^, ^3^

Dermoscopy is a helpful diagnostic tool that has proven useful in BCC, with a sensitivity of 91% and a specificity of 95%.^4^, ^5^ This favors earlier detection and treatment planning; still, histological confirmation remains the gold standard for its diagnosis.^6^, ^7^, ^8^, ^9^ Nowadays, smaller BCCs are found more frequently, probably because of preventive care culture and technological advances. Small BCCs have been defined by some authors as those measuring less than 5 mm, while BCCs lower than 3 mm have been denominated very small BCCs.^10^, ^11^, ^12^, ^13^, ^14^, ^15^ Regarding clinical presentation, small BCCs are more prevalent in younger men, prevail on the face, are often pigmented, and their most common histological subtype is nodular.^11^ Detection of initial tumoral stages is related to an easier treatment, with better cosmetic outcomes and less functional impact.^10^ Today, literature is clear on classic and non-classic dermoscopic criteria of BCC; however, few studies on dermoscopy of small BCCs have been performed, reporting that these BCCs can present both classic and non-classic dermoscopic features, with blue-gray dots and ovoid nests as main structures.^11^, ^12^

Very small BCCs (vsBCCs) are even more incipient lesions, that could display different features due to an early phase of tumoral growth.^14^, ^15^, ^16^, ^17^, ^18^ Dermoscopy on these tumors has not been widely studied, therefore, there is a scarcity of medical literature reporting and comparing dermoscopic characteristics with larger BCCs. The aim of this study was to describe and compare dermoscopic features of BCCs measuring ≤3 mm, with those from 3 to 10 mm in diameter.

## Materials and methods

This was an analytical cross-sectional study conducted between January 2017 and December 2021 in Medellín, Colombia. Inclusion criteria comprehended patients with a histopathological diagnosis of BCC, who had dermoscopic pictures with 10× or 20× magnification. Cases of recurrent BCC and cases without patients consent for photographic analysis were excluded. Cases were retrospectively and prospectively selected from a Skin Cancer Referral Center. An institutional database with all diagnosed BCCs was examined, and cases that met eligibility criteria were included. Prospectively, new cases of histopathological confirmed BCC were examined and if eligible, included. The study size was determined by the total number of BCCs that met eligibility criteria and were diagnosed during the mentioned period. The study protocol was in concordance with the ethical guidelines of the Declaration of Helsinki. Institutional Review Board approval was obtained from Universidad Pontificia Bolivariana.

Two of the researchers were responsible for data and dermoscopic picture recollection. Two databases were created using Microsoft Excel and Microsoft PowerPoint, where information and pictures were organized. Dermoscopy was performed using a polarized device (DermLite DL4, 3 Gen, LLC, San Juan Capistrano, California, United States of America – FotoFinder ATBM, Bad Birnbach, Germany) and dermoscopic images were acquired with a high-resolution camera adapted to the dermoscopic device. Pictures were assessed by two independent observers for predefined dermoscopic features. In case of discordance, a third evaluation was made, and a consensus was achieved. Tumoral size was measured digitally, based on the largest diameter. BCCs were classified according to their size in three groups: 1) vsBCCs (measuring less than 3 mm), 2) Small BCCs (from 3 to 5 mm), and 3) Medium BCCs (from 5 to 10 mm). The latter two were grouped as reference or control BCCs (from 3 up to 10 mm) when compared to vsBCCs.

Demographic, clinical, histopathological and therapeutic features were obtained from medical records and pathology reports and included: age, gender, occupation, personal history of Non-Melanoma Skin Cancer (NMSC) or melanoma, Fitzpatrick skin phototype, signs of local photodamage, number of lesions per patient, size and location of the tumor, specific sublocation in case of facial tumors, primary lesion, predominant color (defined as color present in more than half of the lesion), the person that detected the lesion, time of evolution, histopathological subtype, tumoral depth, clinicopathological concordance, treatment modality, and in case of Mohs Micrographic Surgery (MMS) number of stages, histological subtype on surgery, and concordance with previous biopsy.

The following BCC dermoscopic criteria were analyzed: 1) Lack of pigmented network; 2) Vessels: arborizing vessels, Short-Fine Telangiectasias (SFT), hairpin, corkscrew, glomerular, dotted, comma and lineal irregular vessels, polymorphous vessels (defined as two or more morphologies), milky-red areas and red globules; 3) Pigmented structures: leaf-like structures, spoke-wheel-like structures, blue-gray globules, ovoid nests, concentric structures, and blue-gray dots; 4) Shiny White Structures (SWS): blotches and strands, rosettes, and streaks (orthogonal structures); 5) Scales: white or yellow; and 6) Other structures: ulceration, micro-erosions, multiple aggregated yellow globules (MAY globules), blue-whitish veil, and milia-like cysts.

Quantitative variables were presented as mean and Standard Seviations (SD), according to the normal distribution of variables evaluated with the Kolmogorov-Smirnov test. In the case of non-normality, medians and interquartile ranges were obtained. The size was the dependent variable, classified into three groups, as mentioned before. Dermoscopic features were compared between groups with the statistical tests Chi-Square or Fisher test, depending on the expected value (statistical significance, p < 0.05). The database was exported to IBM SPSS Statistics 24.0 software for statistical analysis.

## Results

### Demographic features

196 patients were included, with a total of 326 BCCs. Of all patients, 60% were male (117/196). The median age was 66.5 years (IQR 56–76); 71% (140/196) had a single lesion, and only 8% (16/196) had four or more lesions. Chronic sun exposure was present in 18% of the patients (36/196). Most of the cases had a personal history of NMSC (61%‒119/196) while a minority had a history of melanoma (7%‒14/196). The most common skin phototype was Fitzpatrick type III, found in 59% (116/196) of the patients. Other features are described in more detail in Table 1. There were no statistically significant differences between very small and reference BCCs on demographic features.

**Table 1:** Demographic characteristics

	N = 196
Feature	n (%)
Sex	
Male	117 (59.7)
Age, years – Median (P25‒P75)	66.5 (56‒76)
Occupation	
Without chronic sun exposure	129 (65.8)
Chronic sun exposure	36 (18.4)
Unknown	31 (15.8)
Lesions per patient	
1	140 (71.4)
2‒3	40 (20.4)
≥4	16 (8.2)
Personal history	
Non-Melanoma Skin Cancer (NMSC)	119 (60.7)
Melanoma	14 (7.1)
Fitzpatrick skin phototype	
I	6 (3.0)
II	56 (28.6)
III	116 (59.2)
IV	18 (9.2)

### Clinical, pathological, and therapeutic features

Frequencies were determined based on all the observed lesions (326). The primary lesion was a non-ulcerated papule in 48% (156/326) of the tumors. Pink was the predominant color in 46% (149/326), followed by brown and red. The brown color was more prevalent in vsBCCs (39% vs. 16% on reference BCCs), without statistical significance, while pink as the main color was more common when size increased (33% vs. 50%). Local photodamage signs were found in 81% (263/326) of the lesions. Lesions were detected by dermatologists in 81% of the cases, while patients detected more frequently control than vsBCCs. Only 12% (40/326) of the tumors had a known evolution time, from 1 to 24 months. Diagnostic impression was BCC in 98% (320/326), even in vsBCCs.

Tumors had a median size of 5 mm (IQR 3.375‒7). vsBCCs represented 25% of the cases (81/326), 39% of lesions measured from 3 to 5 mm (127/326) and 36% from 5 to 10 mm (118/326). The latter two accounted for the reference BCCs group 75% (245/326). The most frequent location was face/neck, in 53% (174/326). On vsBCCs it was even more common, in 69%; additionally, as tumoral size increased, extrafacial locations were more common (p = 0.01). Leading facial lesion locations were nose 28% (45/160), cheek 26% (41/160), and forehead (26%), without statistically significant difference between size groups (p = 0.16).

Prevailing histological subtypes were nodular in 78% (255/326), superficial in 26% (83/326) and micronodular in 6% (21/326). The nodular pattern was found in 93% (75/81) of vsBCCs vs. 74% (180/245) of reference BCCs (p < 0.001); conversely, a superficial pattern was present in 9% (7/81) of vsBCCs vs. 31% (76/245) of control BCCs (p < 0.001). Aggressive histological patterns had similar frequencies in both groups. Most of the lesions had a depth of 1 to 2 mm (70%), still, very small tumors were less profound (p = 0.07). For more details on clinic-pathological features refer to Table 2.

**Table 2:** Clinical and pathological features

	N = 326
Feature	n (%)
*Clinical*	
Size, mm	
≤3	81 (24.8)
>3 – 5	127 (39)
>5 – 10	118 (36.2)
Location	
Face and neck	174 (53.4)
Limbs	67 (20.5)
Torso	60 (18.4)
Scalp	25 (7.7)
Signs of local photodamage	263 (80.7)
Primary lesion	
Non-ulcerated papule	156 (47.9)
Non-ulcerated plaque	142 (43.6)
Ulcerated papule	14 (4.3)
Ulcerated plaque	5 (1.5)
Macule	6 (1.8)
Predominant color	
Pink	149 (45.7)
Brown	69 (21.2)
Red	52 (15.9)
Skin-colored	22 (6.7)
Blue	16 (4.9)
Gray	6 (1.9)
Black	6 (1.9)
White	5 (1.5)
Yellow	1 (0.3)
*Pathology*	
Histological subtype^a^	
Nodular	255 (78.2)
Superficial	83 (25.5)
Micronodular	21 (6.4)
Infiltrative	7 (2.1)
Morpheaform	2 (0.6)
Metatypical	2 (0.6)
Adenoid	2 (0.6)
Keratotic	1 (0.3)
Depth, mm	50 (15.3)
≤1	227 (69.7)
>1–2	31 (9.5)
>2	50 (15.3)
Not reported	18 (5.5)

a Variables are not mutually excluding.

Regarding treatment, 42% (138/326) received MMS, 24% (77/326) underwent conventional resection, 3% (11/326) were resected without a prior biopsy due to a highly suggestive image and specific scenarios, and 31% (100/326) had not received treatment at the end of data recollection. Of those who underwent MMS (138), nodular subtype was documented in 87% (120/138), superficial in 21%, micronodular in 7%, infiltrative in 6% and metatypical in 1%. A total of 2 stages were required in 88% (122/138). Nonetheless, no statistically significant differences were found regarding therapeutic characteristics.

### Dermoscopic features

The pigment network was not identified in any lesion. The five more prevalent structures were: SFT, found in 62% (203/326) followed by blue-gray dots 57% (186/326), SWS blotches and strands 56% (184/326), arborizing vessels 38% (124/326) and blue-gray globules 24% (78/326).

Vessels were identified in 83% (271/326) and were significantly less prevalent in vsBCCs 68% (55/81) than in reference BCCs 88% (216/245) (p < 0.001). Arborizing vessels were found in 38% of all tumors (124/326), without significant differences between groups. SFT was the most common morphology, found in 62% (203/326) of the lesions, and was significantly less frequent in vsBCCs 52% (42/81) than in reference BCCs 66% (161/245) (p = 0.02); nevertheless, they were the most common vessel morphology in the former group. Less common morphologies included hairpin, glomerular, dotted, linear irregular, comma, and corkscrew vessels; polymorphous vessels were present in 21% (67/326) of the lesions. None of the latter showed statistically significant differences.

Pigmented structures were found in 68% (221/326) and were more frequent in vsBCCs 83% (67/81) than in reference groups 63% (154/245) (p = 0.001). Blue-gray dots were the most common pigmented structure 57% (186/326) and were also statistically more prevalent in vsBCCs (67% vs. 54%) (p = 0.04). Other pigmented features found were, in descending order: blue-gray globules, ovoid nests, leaf-like structures, spoke-wheel-like structures, and finally, concentric structures.

SWS, especially blotches, and strands, were significantly less frequent in vsBCCs 27% (22/81) than in reference tumors 66% (162/245) (p < 0.001). Scales were also less common in vsBCCs (11% vs. 31%) (p < 0.001), as were ulcerations and micro-erosions. Further details can be consulted in Table 3.

**Table 3:** Dermoscopic features of very small BCCs vs reference BCCs

	Any size	≤3 mm	3–10 mm	
	N = 326	N = 81	N = 245	
Feature	n (%)	n (%)	n (%)	p-value
Vessels	271 (83.1)	55 (67.9)	216 (88.2)	<0.001
Short-fine telangiectasias	203 (62.3)	42 (51.9)	161 (65.7)	0.02
Arborizing	124 (38)	27 (33.3)	97 (39.6)	0.3
Polymorphous	67 (20.6)	13 (16)	54 (22)	0.2
Hairpin	39 (12)	7 (8.6)	32 (13.1)	0.2
Glomerular	34 (10.4)	5 (6.2)	29 (11.8)	0.1
Dotted	21 (6.4)	4 (4.9)	17 (6.9)	0.5
Linear irregular	18 (5.5)	7 (8.6)	11 (4.5)	0.1
Comma	9 (2.8)	3 (3.7)	6 (2.4)	0.6
Corkscrew	4 (1.2)	1 (1.2)	3 (1.2)	1
Pigmented structures	221 (67.8)	67 (82.7)	154 (62.9)	0.001
Blue-gray dots	186 (57.1)	54 (66.7)	132 (53.9)	0.04
Blue-gray globules	78 (23.9)	24 (29.6)	54 (22)	0.1
Ovoid nests	64 (19.6)	19 (23.5)	45 (18.4)	0.3
Leaf-like structures	54 (16.6)	9 (11.1)	45 (18.4)	0.1
Spoke-wheel-like structures	46 (14.1)	9 (11.1)	37 (15.1)	0.3
Concentric structures	44 (13.5)	6 (7.4)	38 (15.5)	0.06
Shiny white structures	188 (57.7)	23 (28.4)	165 (67.3)	<0.001
Blotches, strands	184 (56.4)	22 (27.2)	162 (66.1)	<0.001
Rosettes	20 (6.1)	2 (2.5)	18 (7.3)	0.1
Streaks, orthogonal	3 (0.9)	0 (0)	3 (1.2)	1
Scales	85 (26.1)	9 (11.1)	76 (31)	<0.001
White	75 (23)	8 (9.9)	67 (27.3)	0.001
Yellow	18 (5.5)	1 (1.2)	17 (6.9)	0.053
Other structures				
Ulceration	42 (12.9)	3 (3.7)	39 (15.9)	<0.001
Micro-erosions	37 (11.3)	2 (2.5)	35 (14.3)	0.004
MAY globules	25 (7.7)	4 (4.9)	21 (8.6)	N/A
Blue-whitish veil	1 (0.3)	0 (0)	1 (0.4)	N/A

When comparing classic BCC features between very small and small BCCs, blue-gray globules and ovoid nests were more frequent in the former. Respecting non-classic features, blue-gray dots were more common in very small lesions, while the rest of features were less frequent in this group (Table 4).

**Table 4:** Dermoscopic classic and non-classic features of very small, small, and medium BCCs

	Any size	≤3 mm	3–5 mm	5–10 mm
	N = 326	N = 81	N = 127	N = 118
Feature	n (%)	n (%)	n (%)	n (%)
*Classic features*				
Arborizing	124 (38)	27 (33.3)	48 (37.8)	49 (41.5)
Blue-gray globules	78 (23.9)	24 (29.6)	30 (23.6)	24 (20.3)
Ovoid nests	64 (19.6)	19 (23.5)	22 (17.3)	23 (19.5)
Leaf-like structures	54 (16.6)	9 (11.1)	27 (21.3)	18 (15.3)
Spoke-wheel-like structures	46 (14.1)	9 (11.1)	19 (15)	18 (15.3)
Ulceration	42 (12.9)	3 (3.7)	14 (11)	25 (21.2)
*Non-classic features*				
Short-fine telangiectasias	203 (62.3)	42 (51.9)	81 (63.8)	80 (67.8)
Blue-gray dots	186 (57.1)	54 (66.7)	77 (60.6)	55 (46.6)
SW blotches and strands	184 (56.4)	22 (27.2)	73 (57.5)	89 (75.4)
Concentric structures	44 (13.5)	6 (7.4)	20 (15.7)	18 (15.3)
Micro-erosions	37 (11.3)	2 (2.5)	8 (6.3)	27 (22.9)
MAY globules	25 (7.7)	4 (4.9)	11 (8.7)	10 (8.5)

**Figure 1:**
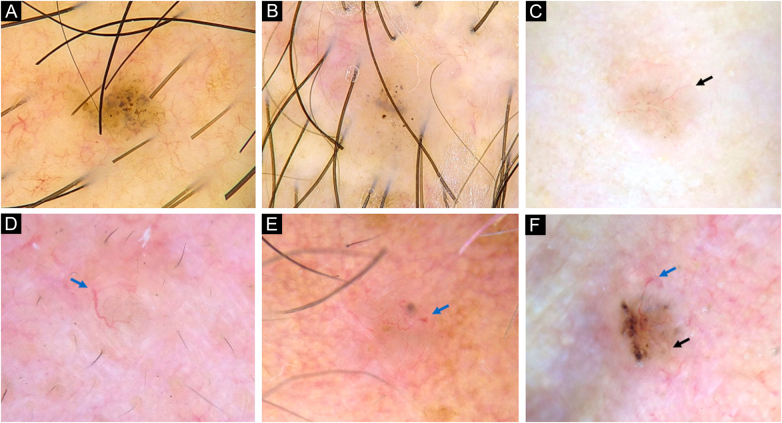
Dermoscopy of very small BCCs. (A) 2 mm BCC located on the chest, displaying blue-gray dots and globules. (B) 2.5 mm BCC located on the scalp, presenting blue-gray dots and globules(C) 2 mm BCC located on the nasal dorsum, blue-gray dots and SFT (black arrow) were identified. (D) 2 mm BCC located on the nasolabial fold, with presence of blue-gray dots and arborizing vessels (blue arrow). (E) 2 mm BCC located on the preauricular region, displaying blue-gray globules, arborizing vessels (blue arrow) and SWS blotches and strands. (F) 2.5 mm BCC located on the inner canthus, with presence of blue-gray dots, globules, SFT (black arrow) and arborizing vessels (blue arrow)

**Figure 2:**
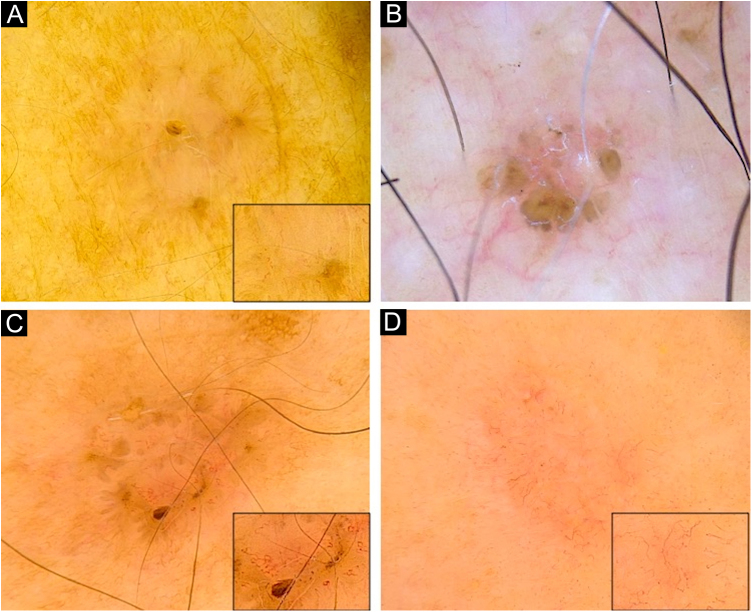
Dermoscopy of superficial very small BCCs. (A) 3 mm BCC located on the upper limb, displaying blue-gray dots, polymorphous vessels (magnified on square) with hairpin, comma, glomerular and linear irregular vessels, and SWS blotches. (B) 3 mm BCC located on the chest, displaying leaf-like structures, ovoid nests, micro-erosions and white scales. (C) 3 mm BCC located on the upper limb, showing blue-gray dots and globules, leaf-like structures, polymorphous vessels (magnified on square) with SFT, hairpin and comma vessels, and SWS blotches. (D) 3 mm BCC located on the upper limb, presenting blue-gray dots, polymorphous vessels (magnified on square) with SFT and hairpin vessels, and SWS blotches

**Figure 3:**
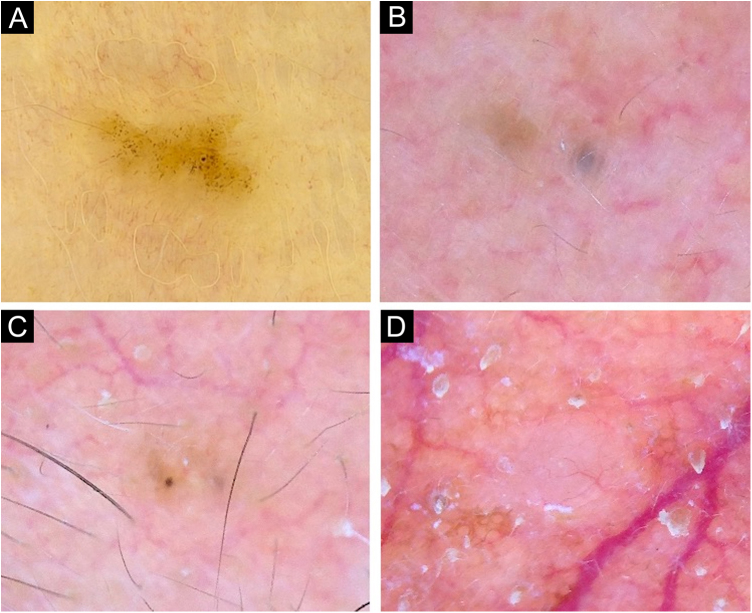
Very small BCCs presenting a single dermoscopic feature. (A) 3 mm BCC located on the upper limb, displaying blue-gray dots. (B) 2 mm BCC located on the forehead, presenting blue-gray globules. (C) 1.5 mm BCC located on the forehead, a single spoke-wheel-like structure was identified. (D) 2 mm BCC located on the alar groove, with presence of arborizing vessels

**Figure 4:**
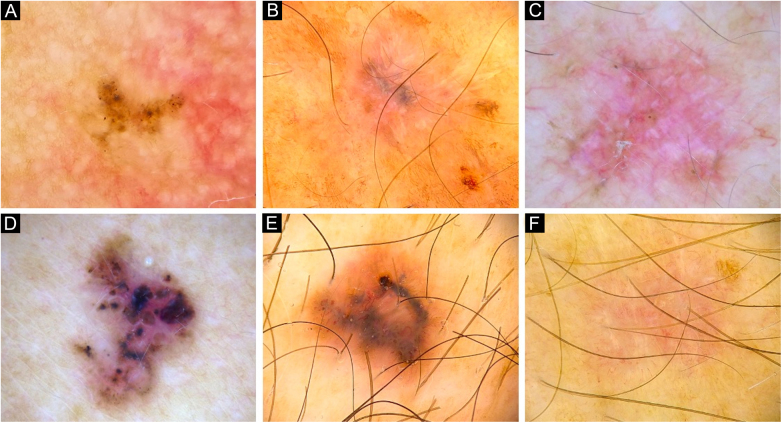
Dermoscopy of reference BCCs. (A) Small BCC of 4 mm of diameter located on the retroauricular region, displaying concentric structures, blue-gray dots and globules. (B) Small BCC of 4.5 mm of diameter located on the upper limb, presenting blue-gray dots, SFT and SWS blotches and strands. (C) Medium BCC measuring 8.5 mm, located on the upper limb, with presence of blue-gray globules and dots, SFT, micro-erosions, SWS blotches, strands and streaks. (D) 10 mm BCC located on the back, with numerous features: spoke-wheel-like structures, concentric structures, blue-gray dots, globules, ovoid nests, polymorphous vessels with SFT, dotted and glomerular vessels, and SWS blotches and strands. (E) 5.5 mm BCC located on the back, presenting leaf-like structures, ovoid nests, blue-gray dots, polymorphous vessels with hairpin, glomerular and lineal irregular vessels. (F) 6.5 mm BCC located on the upper limb, displaying polymorphous vessels with SFT, hairpin and glomerular vessels, SWS blotches and strands, and white scale

vsBCCs were characterized by the presence of blue-gray dots (67%), and occasionally SFT (52%) (Figure 1, Figure 2). The former had a diffuse distribution in 43% (23/54) and was clustered in 41% (22/54). Blue-gray globules and ovoid nests were present on 30% and 24% of these lesions while arborizing vessels were found on 33%. SWS, scales, ulceration, and micro-erosions were rare on them. Just 12% (10/81) presented an isolated dermoscopic feature, usually dots or arborizing vessels 30% (3/10, each one) (Fig. 3); two features were displayed by 20% (16/81), in which dots with SFT or globules were the most common combinations (3/16 ‒ 19%, each one). Moreover, 68% had three or more features. In 84% of very small lesions dermoscopic chaos was noticeable (68/81). Dermoscopic pictures of vsBCCs are depicted in Figure 1, Figure 2, Figure 3, and images of reference BCCs are portrayed in Fig. 4. For further information, please refer to Supplementary Tables (S1‒S5).

## Discussion

Dermoscopy has a high accuracy and precision in the diagnosis of BCC, which benefits an earlier suspicion of malignancy and favors the detection of smaller tumors. In this study, the authors assessed 326 BCCs of 196 patients. vsBCCs accounted for 81 of the tumors. They often had brown as the predominant color (39%) and showed commonly a nodular subtype on histopathology (93%) and rarely a superficial pattern (9%). Interestingly, aggressive subtypes had similar frequencies regardless of tumoral size. Most of the smaller lesions were located on the face and neck, in concordance with current literature;^19^, ^20^ besides, as size increased, prevalence in other areas rose as well. These lesions were more likely to receive MMS, practiced in 48% of vsBCCs vs. 40% of reference BCCs; in 95% (37/39) of vsBCCs who underwent MMS it took more than 1 stage to obtain negative surgical margins.

On dermoscopy of vsBCCs, the presence of pigmented structures was the most frequent dermoscopic characteristic (83%), mainly blue-gray dots (67%), and was more frequent in these tumors when compared with larger BCCs. Conversely, vessels were less frequent in vsBCCs, notably SFT, as were SWS, scales, ulcerations, and micro-erosions. The most common vascular morphologies were SFT (52%) and arborizing vessels (33%). Of lesions displaying SFT, just 7% (3/42) corresponded to superficial histological subtype, 2% (1/42) to mixed subtype micronodular and infiltrative, and 91% (38/42) of them were nodular. This means that SFTs, in this study, were not present in the context of a superficial BCC.

On nodular vsBCCs (75/81) blue-gray dots were also the most frequent feature found in 68% (51/75). Vessels were found in 65% (49/75) of the tumors, mainly SFT in 51% (38/75), and arborizing vessels in 35% (26/75). High-risk histological subtypes were found in 6 very small lesions. Most of them displayed vessels as main dermoscopic feature (83%), mainly arborizing vessels (67%), followed by SFT (50%). Interestingly, in these tumors, blue-gray dots were not as common (33%).

On superficial vsBCCs (7/81), the most common feature was also the presence of pigmented structures in 86% (6/7), mainly blue-gray dots (57%), followed by ovoid nests and leaf-like structures (43% each). Vessels were found in 71% (5/7) of these lesions, and, interestingly, were polymorphous in all the cases, with glomerular morphology predominating 57% (4/7), followed by SFT 43% (3/7); however, arborizing vessels were not identified. Just one of these lesions presented micro-erosions.

Takahashi et al.^10^ published a study comparing BCCs ≤3 mm (n = 6) with BCCs from 4 to 6 mm (n = 11). Blue-gray dots and globules were present on 100% of the cases of the former group, and nests on 67%, in contrast to the present study, where they were found on 67%, 30%, and 24% of vsBCCs, respectively. Additionally, they found just 9% of lesions had blue-gray dots in the group from 4 to 6 mm, contrary to the results, which showed they were present on 54% of reference BCCs. The main limitation of their study was the small size of the sample.

Sanchez-Martin et al.^12^ published a study on 100 BCCs < 5 mm, divided into two groups, comparing vsBCCs (n = 34) with small BCCs (n = 66). The most frequent structure was SFT (78%), followed by blue-gray dots (55%). However, they did not find any statistically significant difference between very small and small BCCs, differing from the present results (Table 4).

Longo et al.^13^ compared BCCs <5 mm with control lesions. They found small BCCs were more prevalent in younger patients, in contrast to the present results, since the authors did not find any association between age and tumoral size. The nodular pattern was also the more frequent subtype in their study, with an odds ratio of 3.4. They found two dermoscopic predictors of small BCCs: blue-gray dots and ovoid nests, however, these features had weak odds ratios and their confidence interval crossed the unity in both cases. Almost all dermoscopic features were observed equally in both groups, and the only statistically significant difference was a lower frequency of ulceration and micro-erosions in BCCs of <5 mm.

Di Meo et al.^11^ collected 100 BCCs of <5 mm from a database and evaluated global concordance between observers with low dermoscopic experience. They found a low level of concordance for some of the classic BCC criteria, and no concordance for almost all non-classic criteria. As seen in the present study, the presence of blue-gray dots, a non-classic feature, is key to detecting incipient lesions. This confirms the importance of always evaluating both classic and non-classic criteria, since the latter may correlate with an early tumoral stage.

The present study has some limitations. Cases were collected from a Skin Cancer Reference Center, which may alter patients’ demographic characteristics. Since it was conducted in Colombia, most phototypes ranged from I to IV, with less data on clearer and darker skin phototypes. It remains to be confirmed in larger studies with long-term follow-up, if early detection of very small lesions improves with dermoscopy, and the prognostic value that it may have.

## Conclusion

In conclusion, on dermoscopy of vsBCCs (≤3 mm), pigmented structures are more frequent, especially blue-gray dots, while vessels and SWS are less prevalent. Thus, when evaluating a lesion smaller than 3 mm and suspicious for BCC, blue-gray dots and pigmented features must be actively sought, as its presence correlates with BCC. This study has robust evidence that may favor an earlier diagnosis in initial tumoral growth stages, rising the probability of acceptable better and functional outcomes after treatment.

## Financial support

None declared.

## Authors' contributions

Camilo Arias-Rodriguez: Study concept and design; data collection, analysis and interpretation; writing of the manuscript; critical review of the literature; final approval of the final version of the manuscript.

Ana Maria Muñoz-Monsalve: Study concept and design; effective participation in the research guidance; critical review of important intellectual content; final approval of the final version of the manuscript.

Diana Cuesta: Study concept and design; data analysis and interpretation; writing of the manuscript; critical review of the literature; final approval of the final version of the manuscript.

Susana Mejia-Mesa: Study concept and design; data analysis and interpretation; writing of the manuscript; critical review of the literature; final approval of the final version of the manuscript.

Maria Soledad Aluma-Tenorio: Study concept and design; data analysis and interpretation; writing of the manuscript; critical review of the literature; final approval of the final version of the manuscript.

## Conflicts of interest

None declared.
