# Supplementary material for: Dermoscopy of very small basal cell carcinoma (≤3 mm)
Source: An Bras Dermatol. 2023 Jul 6;98(6):755–63. doi: 10.1016/j.abd.2022.12.004 (PMC10589476; doi:10.1016/j.abd.2022.12.004)
Supplement: Supplementary file 1 [file mmc1.doc]

**Supplementary material**

**Table S1.** Location according to size

| Location | *Any size*  *N =* 326  *n* (%) | ≤ 3mm  *N =* 81  *n* (%) | 3 – 10mm  *N =* 245  *n* (%) |
| --- | --- | --- | --- |
| Face and neck  Limbs  Torso  Scalp  P value = 0.01 | 174 (53.4)  67 (20.6)  60 (18.4)  25 (7.7) | 56 (69.1)  10 (12.3)  11 (13.6)  4 (4.9) | 118 (48.2)  57 (23.3)  49 (20)  21 (8.6) |

**Table S2.** Location of facial lesions according to size

| Location | *Any size*  *N =* 160  *n* (%) | ≤ 3mm  *N =* 52  *n* (%) | 3 – 10mm  *N =* 108  *n* (%) |
| --- | --- | --- | --- |
| Nose  Forehead  Cheek  Periauricular  Periorbital  Perioral  P value = 0.166 | 45 (28.1)  41 (25.6)  41 (25.6)  14 (8.8)  13 (8.1)  6 (3.8) | 17 (32.7)  12 (23.1)  11 (21.2)  3 (5.8)  8 (15.4)  1 (1.9) | 28 (25.9)  29 (26.9)  30 (27.8)  11 (10.2)  5 (4.6)  5 (4.6) |

**Table S3.** Histological subtypes according to size

| Histological subtype | *Any size*  *N =* 326  *n* (%) | ≤ 3mm  *N =* 81  *n* (%) | 3 – 10mm  *N =* 245  *n* (%) | P value |
| --- | --- | --- | --- | --- |
| Nodular  Superficial  Micronodular  Infiltrative  Morpheaform  Metatypical  Adenoid  Keratotic | 255 (78.2)  83 (25.5)  21 (6.4)  7 (2.1)  2 (0.6)  2 (0.6)  2 (0.6)  1 (0.3) | 75 (92.6)  7 (8.6)  5 (6.2)  2 (2.5)  0 (0)  0 (0)  0 (0)  0 (0) | 180 (73.5)  76 (31)  16 (6.5)  5 (2)  2 (0.8)  2 (0.8)  2 (0.8)  1 (0.4) | < 0.001  < 0.001  0.09  1  1  1  1  1 |

**Table S4.** Depth according to size

| Depth, mm | *Any size*  *N =* 308  *n* (%) | ≤ 3mm  *N =* 81  *n* (%) | 3 – 10mm  *N =* 245  *n* (%) |
| --- | --- | --- | --- |
| ≤ 1  > 1 – 2  > 2  P value = 0.07 | 50 (16.2)  227 (73.7)  31 (10.1) | 8 (10.1)  66 (83.5)  5 (6.3) | 42 (18.3)  161 (70.3)  26 (11.4) |

**Table S5.** Dermoscopic features of miniaturized, small and reference BCCs

| Feature | *Any size*  *N =* 326  *n* (%) | ≤ 3mm  *N =* 81  *n* (%) | 3 – 5mm  *N =* 127  *n* (%) | 5 – 10mm  *N =* 118  *n* (%) |
| --- | --- | --- | --- | --- |
| Vessels | 271 (83.1) | 55 (67.9) | 109 (85.8) | 107 (90.7) |
| Short-fine telangiectasias  Arborizing  Polymorphous  Hairpin  Glomerular  Dotted  Linear irregular  Comma  Corkscrew  Pigmented structures  Blue-gray dots  Blue-gray globules  Ovoid nests  Leaf-like structures  Spoke-wheel-like structures  Concentric structures  Shiny white structures  Blotches, strands  Rosettes  Streaks, orthogonal  Scales  White  Yellow  Other structures  Ulceration  Micro-erosions  MAY globules  Blue-whitish veil | 203 (62.3)  124 (38)  67 (20.6)  39 (12)  34 (10.4)  21 (6.4)  18 (5.5)  9 (2.8)  4 (1.2)  221 (67.8)  186 (57.1)  78 (23.9)  64 (19.6)  54 (16.6)  46 (14.1)  44 (13.5)  188 (57.7)  184 (56.4)  20 (6.1)  3 (0.9)  85 (26.1)  75 (23)  18 (5.5)  42 (12.9)  37 (11.3)  25 (7.7)  1 (0.3) | 42 (51.9)  27 (33.3)  13 (16)  7 (8.6)  5 (6.2)  4 (4.9)  7 (8.6)  3 (3.7)  1 (1.2)  67 (82.7)  54 (66.7)  24 (29.6)  19 (23.5)  9 (11.1)  9 (11.1)  6 (7.4)  23 (28.4)  22 (27.2)  2 (2.5)  0 (0)  9 (11.1)  8 (9.9)  1 (1.2)  3 (3.7)  2 (2.5)  4 (4.9)  0 (0) | 81 (63.8)  48 (37.8)  29 (22.8)  24 (18.9)  12 (9.4)  5 (3.9)  8 (6.3)  3 (2.4)  1 (0.8)  87 (68.5)  77 (60.6)  30 (23.6)  22 (17.3)  27 (21.3)  19 (15)  20 (15.7)  75 (59.1)  73 (57.5)  7 (5.5)  0 (0)  31 (24.4)  28 (22)  4 (3.1)  14 (11)  8 (6.3)  11 (8.7)  0 (0) | 80 (67.8)  49 (41.5)  25 (21.2)  8 (6.8)  17 (14.4)  12 (10.2)  3 (2.5)  3 (2.5)  2 (1.7)  67 (56.8)  55 (46.6)  24 (20.3)  23 (19.5)  18 (15.3)  18 (15.3)  18 (15.3)  90 (76.3)  89 (75.4)  11 (9.3)  3 (2.5)  45 (38.1)  39 (33.1)  13 (11)  25 (21.2)  27 (22.9)  10 (8.5)  1 (0.8) |
